# *TRMT10C* gene polymorphisms confer hepatoblastoma susceptibility: evidence from a seven-center case-control study

**DOI:** 10.7150/jca.98555

**Published:** 2024-08-19

**Authors:** Yanfei Liu, Jinhong Zhu, Xianqiang Wang, Wenli Zhang, Yong Li, Zhonghua Yang, Jiao Zhang, Jiwen Cheng, Li Li, Suhong Li, Jing He, Jun Bian

**Affiliations:** 1 Department of Pediatric Surgery, Xi'an Children's Hospital, Xi'an Jiaotong University Affiliated Children's Hospital, Xi'an 710003, Shaanxi, China.; 2 Department of Clinical Laboratory, Biobank, Harbin Medical University Cancer Hospital, Harbin 150040, Heilongjiang, China.; 3 Department of General Pediatrics, Senior Department of Pediatrics, National Engineering Laboratory for Birth Defects Prevention and Control of Key Technology, Beijing Key Laboratory of Pediatric Organ Failure, the Seventh Medical Center of PLA General Hospital, Beijing 100000, China.; 4 Department of Pediatric Surgery, Guangzhou Institute of Pediatrics, Guangdong Provincial Key Laboratory of Research in Structural Birth Defect Disease, Guangzhou Women and Children's Medical Center, Guangzhou Medical University, Guangzhou 510623, Guangdong, China.; 5 Department of Pediatric Surgery, Hunan Children's Hospital, Changsha 410004, Hunan, China.; 6 Department of Pediatric Surgery, Shengjing Hospital of China Medical University, Shenyang 110004, Liaoning, China.; 7 Department of Pediatric Surgery, the First Affiliated Hospital of Zhengzhou University, Zhengzhou 450052, Henan, China.; 8 Department of Pediatric Surgery, the Second Affiliated Hospital of Xi'an Jiaotong University, Xi'an 710004, Shaanxi, China.; 9 Kunming Key Laboratory of Children Infection and Immunity, Yunnan Key Laboratory of Children's Major Disease Research, Yunnan Institute of Pediatrics Research, Yunnan Medical Center for Pediatric Diseases, Kunming Children's Hospital, Kunming 650228, Yunnan, China.; 10 Department of Pathology, Children Hospital and Women Health Center of Shanxi, Taiyuan 030013, Shannxi, China.

**Keywords:** *TRMT10C*, m^1^A modification, polymorphism, hepatoblastoma, susceptibility

## Abstract

N1-methyladenosine (m^1^A) is a reversible epigenetic modification of RNAs. Aberrant m^1^A modification levels due to dysregulation of m^1^A regulators have been observed in multiple cancers. tRNA methyltransferase 10C (TRMT10C) can install m^1^A in RNAs; however, its role in hepatoblastoma remains unknown. We conducted this study to identify causal polymorphisms in the *TRMT10C* gene for hepatoblastoma susceptibility in a cohort of Chinese children (313 cases vs. 1446 controls). The genotypes of four potential functional polymorphisms (rs7641261 C>T, rs2303476 T>C, rs4257518 A>G, and rs3762735 C>G) were determined in participants using TaqMan real-time PCR. The associations of these polymorphisms with hepatoblastoma susceptibility were estimated by logistic regression analysis adjusted for age and sex. All four polymorphisms were significantly associated with hepatoblastoma risk. In particular, under the recessive genetic model, these polymorphisms conferred an increased risk of hepatoblastoma: rs7641261 C>T [adjusted odds ratio (OR)=1.64, 95% confidence interval (CI)=1.04-2.58, *P*=0.033], rs2303476 T>C (adjusted OR=1.87, 95% CI=1.16-3.02, *P*=0.010), rs4257518 A>G (adjusted OR=1.45, 95% CI=1.09-1.94, *P*=0.012), and rs3762735 C>G (adjusted OR=3.83, 95% CI=2.15-6.82, *P*<0.0001). Combined analysis revealed that kids had an increased risk of developing hepatoblastoma if they harbored at least one risk genotype (adjusted OR=1.94, 95% CI=1.48-2.54, *P*<0.0001). In addition, the combined risk effects of the four SNPs persisted across all the subgroups. We identified four hepatoblastoma susceptibility loci in the *TRMT10C* gene. Identifying more disease-causing loci may facilitate the development of genetic marker panels to predict individuals' hepatoblastoma predisposition.

## Introduction

N1-methyladenosine (m^1^A), a methyl group in the first nitrogen atom of adenosine, is a reversible epigenetic modification in mRNAs, tRNAs, rRNAs, and lncRNAs 1. Positive electrostatic charge-carrying m^1^A modifications interfere with A:U base pairs and therefore affect the processing, structure, stability, translation, and functions of host RNAs and their interactions with other molecules 2. Accumulating evidence indicates that dysregulation of m^1^A is associated with tumorigenesis, including in ovarian cancer, hepatocellular carcinoma, bladder cancer, gastric cancer, and glioma 3-7. m^1^A modifications in RNAs are finely orchestrated, deposited by methyltransferases (TRMT6, TRMT10C, TRMT61A, TRMT61B, and NML) and eradicated by demethylases (ALKBH1, ALKBH3, ALKBH7, and FTO); these modifications are ultimately sensed by m^1^A-recognizing proteins (YTHDC1 and YTHDF1-3) 1, 5.

tRNA methyltransferase 10C (TRMT10C), also known as the mitochondrial RNase P subunit, is a bifunctional methyltransferase 8. The TRMT10C/MRPP1-HSD17B10/MRPP2 subcomplex installs m^1^A and N^1^-methylguanine (m^1^G) at position 9 in tRNAs 8, 9. TRMT10C/MRPP1 is the catalytic N^1^-methyltransferase subunit 8. Moreover, this complex is also a component of mitochondrial ribonuclease P, which facilitates tRNA maturation 10. Knowledge of the biological roles of this complex is extremely limited. Similar to other m^1^A methyltransferases (e.g., TRMT6 and TRMT61B) 6, 11, 12, a study suggested that TRMT10C may also be involved in carcinogenesis. Knockdown of TRMT10C suppressed the growth and migration of ovarian and cervical cancer cells 13.

Hepatoblastoma is the most frequently diagnosed malignant liver neoplasm in childhood, with an extraordinarily low incidence ranging from 1.2 to 1.5 cases per million people annually 14. The annual incidence rate of hepatoblastoma among Chinese children is estimated to be approximately 1.4 cases per million people 15. Despite reported environmental risk factors, such as inferior birth status, oxygen therapy, radiation, and plasticizers, hepatoblastoma is considered a disease of genetic susceptibility 16, 17. Substantial evidence has suggested the implications of hereditary genetic variants in hepatoblastoma. Several constitutional genetic syndromes, such as trisomy 18/Edward's syndrome, Beckwith-Wiedemann syndrome (BWS), and familial adenomatous polyposis (FAP), predispose patients to hepatoblastoma. Moreover, hepatoblastoma has a lower frequency of somatic mutations than other solid pediatric tumors do 18. In addition, hepatoblastoma susceptibility-related single-nucleotide polymorphisms (SNPs) have been reported in many genes, including genes related to RNA N6-methyladenosine (m^6^A) and N7-methylguanosine (m^7^G) modifications (e.g., *WTAP*, *METTL3*, *METTL14*, *FTO*, *YTHDF1*, *YTHDC1*,* WDRT4*, and *METTL1*) 19-26. However, the associations between genetic polymorphisms in the *TRMT10C* gene and hepatoblastoma risk remain unclear. Our study aimed to identify pathogenic genetic polymorphisms for hepatoblastoma in 313 Chinese children with hepatoblastoma and 1446 age-, sex- and ethnicity-matched healthy controls.

## Materials and Methods

### Patients and study design

The study population included 1759 Han Chinese descendants (313 cases and 1446 controls) under 14 years of age (**Table S1**) 19, 27. Hepatoblastoma was diagnosed in patients by comprehensively considering their clinical symptoms, laboratory test results, pathological reports, and imaging manifestations. Criteria for enrolling hepatoblastoma patients included: 1) Han Chinese lineage, 2) a new diagnosis with histopathological confirmation, 3) no family history of disorders or cancer, and 4) age 14 or younger. Exclusion criteria involved receiving medical intervention or not providing signed informed consent. Sufficient peripheral whole blood samples were obtained from participants for analysis. Patients and healthy controls were recruited from seven independent hospitals in the capitals of Guangdong, Yunnan, Hunan, Shaanxi, Shannxi, Henan, and Liaoning Provinces. Patients were matched to controls according to age and sex. We estimated the clinical stages of patients according to the PRETEXT classification 28. Only children who provided informed consent and sufficient peripheral blood samples free of any treatment were included in this study. The study was evaluated and permitted by the institutional review board of Guangzhou Women and Children's Medical Center (Approval No: 202016601). This study was conducted in accordance with the Declaration of Helsinki.

### Selecting and genotyping polymorphisms

Candidate SNPs in the *TRMT10C* gene were first selected from the dbSNP database (http://www.ncbi.nlm.nih.gov/projects/SNP) on the basis of previously published criteria 29-31: 1) SNPs with a minor allele frequency (MAF) of 5% in the Chinese Han population to ensure sufficient statistical power; 2) SNPs located in exonic regions, promoters and regulatory elements, splice sites, and 5' and 3' UTRs; 3) SNPs with potential functions; and 4) SNPs in low linkage disequilibrium (LD) with each other to avoid redundancy and multiple testing issues. The retrieved SNPs were subsequently entered into the SNPinfo online tool (https://snpinfo.niehs.nih.gov) to filter out SNPs without potential biological functions. For this study, we selected SNPs with low LD, specifically those with R^2^<0.8, according to the web tool LDlink (https://ldlink.nih.gov/?tab=ldmatrix). Four *TRMT10C* gene SNPs (rs7641261 C>T, rs2303476 T>C, rs4257518 A>G, and rs3762735 C>G) were eligible for the study 32. The rs7641261 C>T, rs2303476 T>C, and rs4257518 A>G are located in the transcription factor binding site (TFBS), a region to which a specialized protein binds to increase or decrease the efficiency of gene transcription. rs3762735 C>G is a nonsynonymous SNP that results in a change in the amino acid sequence of a protein, thereby leading to changes in the function and 3D structure of the protein. We used Tiangen Blood DNA Extraction kits (Tiangen Biotechnology, Beijing, China) to recover genomic DNA from subjects' peripheral blood samples. Genotyping assays using TaqMan real-time PCR were executed on a TaqMan instrument (Applied Biosystems, Foster City, CA). For quality control, no-template controls were included in each 384-well plate to check for contamination, along with known genotype controls, to validate the assay performance. Each 384-well plate had at least four positive controls with different genotypes (e.g., homozygous for both alleles and heterozygous). We included technical replicates within the same 384-well plate to assess reproducibility, and we randomly selected 5-10% of the samples per plate for repeat genotyping to verify consistency, aiming for >99% concordance.

### Statistical analysis

The Hardy‒Weinberg equilibrium (HWE) of the SNPs was assessed in the controls using a goodness-of-fit χ^2^ test. We performed logistic regression analysis to calculate odds ratios (ORs) and 95% confidence intervals (CIs) for the associations between selected SNPs and hepatoblastoma susceptibility with the inclusion of adjusted covariates (age and sex). The common and minor alleles of each SNP are denoted W (wild) and M (mutant), respectively. Different genetic models, including homozygous (MM vs. WW), heterozygous (MW vs. WW), dominant (MW/MM vs. WW), and recessive (MM vs. MW/WW) models, were employed to evaluate the associations between SNPs and hepatoblastoma risk in the whole study population. Association studies were also performed for subgroups classified by age, sex, and clinical stage. SAS v9.1 software (SAS Institute Inc., Cary, NC) was used for the two-tailed statistical analyses. *P* values < 0.05 indicated significance.

## Results

### Association of *TRMT10C* gene polymorphisms with hepatoblastoma susceptibility

Age, gender, and clinical information of participants are presented in (**Table S1**). We obtained genotype information on rs7641261 C>T, rs2303476 T>C, rs4257518 A>G, and rs3762735 C>G for 294 cases and 1444 controls. The goodness-of-fit chi-square test confirmed that the genotype frequencies of all the SNPs were distributed according to Hardy‒Weinberg equilibrium in the normal controls (**Table 1**). Compared with the CC genotype, the 7641261 CT genotype conferred a decreased risk of developing hepatoblastoma of 0.36 (adjusted OR=0.64, 95% CI=0.48-0.85, *P*=0.002). Interestingly, the 7641261 C>T polymorphism was associated with decreased hepatoblastoma risk under the dominant genetic model (adjusted OR=0.75, 95% CI=0.57-0.97, *P*=0.029) but increased risk under the recessive genetic model (adjusted OR=1.64, 95% CI=1.04-2.58, *P*=0.033). Similar associations with hepatoblastoma risk were found for rs4257518 A>G under the heterozygous (adjusted OR=0.59, 95% CI=0.44-0.79, *P*=0.0003), dominant (adjusted OR=0.74, 95% CI=0.58-0.96, *P*=0.025), and recessive (adjusted OR=1.45, 95% CI=1.09-1.94, *P*=0.012) genetic models (**Table 1**). The rs2303476 T>C was shown to increase hepatoblastoma risk by 1.83-fold under the homozygous model (adjusted OR=1.83, 95% CI=1.12-2.97, *P*=0.015) and by 1.87-fold under the recessive model (adjusted OR=1.87, 1.16-3.02, *P*=0.010). The opposite results were found for the rs3762735 C>G polymorphism under the heterozygous (adjusted OR=0.51, 95% CI=0.35-0.75, *P*=0.0005), homozygous (adjusted OR=3.42, 95%=1.91-6.11, *P*<0.0001), and recessive (adjusted OR=3.83, 95%=2.15-6.82, *P*<0.0001) genetic models. We next referred to rs7641261 TT, rs2303476 CC, rs4257518 GG, and rs3762735 GG as risk genotypes and found combined effects of the four SNPs on hepatoblastoma risk. The adjusted OR for the subpopulation of children with 1-4 risk genotypes was 1.94 (95% CI=1.48-2.54, *P*<0.0001), indicating the significant deleterious effects of possessing one or more risk genotypes.

### Stratification analysis

Stratification analysis was conducted under the dominant genetic model. The study population was dichotomized by age, sex, and clinical stage. Surprisingly, no significant associations were detected for the rs7641261 polymorphism among all the subgroups (**Table 2**). Moreover, we found a significant association with the rs4257518 polymorphism in boys (OR=0.67, 95%=0.48-0.94, *P*=0.021). Finally, the concurrence of 1-4 risk genotypes was demonstrated to significantly increase hepatoblastoma risk among all subgroups divided by age, sex, and clinical stage. This finding suggests the robustness of the risk effects of the combined SNPs on hepatoblastoma predisposition.

## Discussion

Compared with adult tumors, solid pediatric tumors generally harbor far lower tumor mutation burdens; in particular, hepatoblastoma has the fewest somatic mutations 16, 33, 34. Therefore, heritable genetic variants in cancer predisposition genes play crucial roles in the pathogenesis of hepatoblastoma.

Here, we performed a candidate gene association study to identify hepatoblastoma susceptibility loci in the *TRMT10C* gene. We collected genotype data for four SNPs (rs7641261 C>T, rs2303476 T>C, rs4257518 A>G, and rs3762735 C>G) from 294 cases and 1444 controls. All these SNPs independently modulated hepatoblastoma susceptibility to some extent. Moreover, combination analyses indicated that children with one or more risk genotypes of the four SNPs had a significantly greater risk of developing hepatoblastoma than noncarriers did. Stratification analysis further revealed that the existence of at least one genotype conferred hepatoblastoma susceptibility regardless of age, sex, or clinical stage. The protein product of the *TRMT10C* gene is a tRNA N^1^-methyltransferase, which, coupled with HSD17B10/MRPP2, catalyzes m^1^A modifications in RNAs 9. RNA modifications, including m^6^A, m^1^A, 5-methylcytidine (m^5^C), m^7^G, and 2′-O-methylation (2′-O-Me), are garnering increasing attention in cancer research because of their ability to regulate gene expression and tumorigenesis 35. The role of m^6^A RNA methylation has been investigated most often in cancer, followed by m^7^G 5. Like m^1^A modifications, m^6^A and m^7^G RNA modifications are also dynamically controlled and recognized by regulatory enzymes categorized as writers, erasers, and readers 35. Using the candidate gene method, we verified that these m^6^A and m^7^G regulators are susceptibility genes in several solid pediatric cancers, including glioma 36, 37, neuroblastoma 38, Wilms tumor 39-41, and hepatoblastoma 20-26.

Although less studied, m^1^A modifications are known to regulate diverse cellular functions in carcinogenesis, such as proliferation, invasion, cell metabolism, senescence and cell death, and the tumor microenvironment 42. The TRM6/61 methyltransferase complex can mediate the methylation of adenosine 58 of the initiator methionine tRNA (tRNAi Met) 43. Elevated TRM6/61 expression promotes the anchorage-independent growth and tumor sphere formation of C6 cells (rat glioma cells) by regulating the translation of relevant mRNAs 43. ALKBH3, an m^1^A demethylase of tRNA, is also reported to be oncogenic 44. ALKBH3-mediated m^1^A-demethylated tRNA is vulnerable to angiogenin cleavage, a process that generates apoptosis-preventing tRNA-derived small RNAs (tDRs). Therefore, ALKBH3 can accelerate cancer progression by promoting cancer cell proliferation and invasion 44. However, the role of TRMT10C in cancer has just begun to emerge. To date, only one publication has suggested that TRMT10C is significantly upregulated in CESC, OV, and UCEC and is associated with an unfavorable prognosis in patients with gynecological cancers 13. *In vitro* functional analyses revealed that depletion of TRMT10C inhibited the malignant behaviors of ovarian cancer and cervical cancer cells 13. Consistent with previous association studies with genetic polymorphisms in m^6^A- and m^7^G-related genes, our results indicate that *TRMT10C* is also a hepatoblastoma susceptibility gene. Our results are biologically plausible, although the mechanisms by which *TRMT10C* gene SNPs affect hepatoblastoma susceptibility are unknown.

Ali and colleagues reported that mitochondrial m^1^A/G RNA modification levels are associated with genetic polymorphisms in the tRNA methyltransferase *TRMT61B* gene and the *MRPP3* gene encoding mitochondrial ribonuclease P protein 3, which is responsible for mitochondrial tRNA 5'-end processing 45. Minor alleles of the *MRPP3* missense SNP rs11156878 are significantly associated with increased methylation levels of tRNA P9 45. Moreover, an intronic *TRMT61B* rs11684695 polymorphism was shown to significantly increase RNA2 methylation in blood samples 45. More importantly, the same study demonstrated that the SNPs associated with inferred RNA methylation levels are frequently disease-causing in glaucoma, psoriasis, and breast cancer 45.

It is well documented that potential functional SNPs that may influence expression and function could modify disease susceptibility. The SNPs rs7641261 C>T, rs2303476 T>C, and rs4257518 A>G are positioned in transcription factor binding sites, where they affect *TRMT10C* gene transcription by enabling the binding of particular proteins. On the other hand, rs3762735 C>G is a nonsynonymous SNP that causes an amino acid substitution, impacting both the function and 3D structure of the TRMT10C protein. For instance, the nonsynonymous SNPs C677T (rs1801133) and A1298C (rs1801131) in the *MTHFR* gene reduce the enzyme activity of the gene-encoded protein, 5,10-methylenetetrahydrofolate reductase, consequently affecting non-Hodgkin lymphoma susceptibility 46. Overall, it is reasonable to speculate that the studied SNPs that may alter *TRMT10C* expression and function could modify hepatoblastoma susceptibility by altering the m^1^A modification levels of target RNAs and inducing the crucial effects of m^1^A on fundamental cell processes.

This study has several limitations. First, this study was a retrospective case‒control study, and our conclusions should be confirmed in future prospective studies. Second, the number of cases needs to be expanded. Third, the effects of the significant SNPs on the expression levels and functions of the *TRMT10C* gene should be tested in clinical samples and *in vitro* studies. Fourth, the study cohort was exclusively composed of Han Chinese children, which ensures a homogeneous genetic background and minimizes confounding effects related to ethnic diversity. However, future research should include diverse populations to validate these findings and evaluate their broader applicability. Expanding the study to other ethnic groups will help assess the generalizability of the results and identify potential ethnic-specific genetic or environmental risk factors. Finally, the current analysis does not account for environmental influences, which are critical in disease development. Future investigations should integrate environmental factors, such as exposure to toxins, birth status, and socioeconomic conditions. This comprehensive approach could provide deeper insights into the multifactorial nature of hepatoblastoma susceptibility and enhance our understanding of the interplay between genetics and the environment.

In conclusion, we identified four potential functional SNPs in the *TRMT10C* gene as hepatoblastoma susceptibility loci. The discovery of *TRMT10C* polymorphisms as susceptibility loci may make a valuable contribution to the understanding of genetic risk factors for hepatoblastoma. Our results suggest that the role of TRMT10C in the tumorigenesis of hepatoblastoma should be explored. The discovery of new susceptibility SNPs may help improve hepatoblastoma risk assessment.

## Supplementary Material

Supplementary table.

## Figures and Tables

**Table 1:** Association of *TRMT10C* gene polymorphisms with hepatoblastoma susceptibility.

Genotype	Cases (N=294)	Controls (N=1444)	*P* ^ a^	Crude OR (95% CI)	*P*	Adjusted OR (95% CI) ^b^	*P* ^ b^
rs7641261 C>T (HWE=0.431)							
CC	194 (65.99)	854 (59.14)		1.00		1.00	
CT	73 (24.83)	506 (35.04)		**0.64 (0.48-0.85)**	**0.002**	**0.64 (0.48-0.85)**	**0.002**
TT	27 (9.18)	84 (5.82)		1.42 (0.89-2.24)	0.140	1.42 (0.89-2.25)	0.140
Additive			0.376	0.91 (0.74-1.12)	0.376	0.91 (0.74-1.12)	0.376
Dominant	100 (34.01)	590 (40.86)	0.029	**0.75 (0.57-0.97)**	**0.029**	**0.75 (0.57-0.97)**	**0.029**
Recessive	267 (90.82)	1360 (94.18)	0.031	**1.64 (1.04-2.58)**	**0.033**	**1.64 (1.04-2.58)**	**0.033**
rs2303476 T>C (HWE=0.441)							
TT	180 (61.43)	908 (62.88)		1.00		1.00	
TC	88 (30.03)	468 (32.41)		0.94 (0.71-1.25)	0.681	0.94 (0.71-1.24)	0.664
CC	25 (8.53)	68 (4.71)		**1.84 (1.14-3.00)**	**0.013**	**1.83 (1.12-2.97)**	**0.015**
Additive			0.166	1.16 (0.94-1.42)	0.166	1.15 (0.94-1.42)	0.180
Dominant	113 (38.57)	536 (37.12)	0.641	1.06 (0.82-1.38)	0.641	1.06 (0.82-1.37)	0.666
Recessive	268 (91.47)	1376 (95.29)	0.008	**1.89 (1.17-3.04)**	**0.009**	**1.87 (1.16-3.02)**	**0.010**
rs4257518 A>G (HWE=0.092)							
AA	117 (39.93)	478 (33.10)		1.00		1.00	
AG	98 (33.45)	678 (46.95)		**0.59 (0.44-0.78)**	**0.0003**	**0.59 (0.44-0.79)**	**0.0003**
GG	78 (26.62)	288 (19.94)		1.10 (0.80-1.51)	0.572	1.09 (0.79-1.51)	0.586
Additive			0.974	1.00 (0.84-1.18)	0.974	1.00 (0.84-1.18)	0.957
Dominant	176 (60.07)	966 (66.90)	0.025	**0.74 (0.58-0.96)**	**0.025**	**0.74 (0.58-0.96)**	**0.025**
Recessive	215 (73.38)	1156 (80.06)	0.011	**1.46 (1.09-1.95)**	**0.011**	**1.45 (1.09-1.94)**	**0.012**
rs3762735 C>G (HWE=0.167)							
CC	239 (81.29)	1107 (76.66)		1.00		1.00	
CG	34 (11.56)	308 (21.33)		**0.51 (0.35-0.75)**	**0.0006**	**0.51 (0.35-0.75)**	**0.0005**
GG	21 (7.14)	29 (2.01)		**3.35 (1.88-5.98)**	**<0.0001**	**3.42 (1.91-6.11)**	**<0.0001**
Additive			0.874	1.02 (0.79-1.31)	0.873	1.02 (0.80-1.31)	0.864
Dominant	55 (18.71)	337 (23.34)	0.083	0.76 (0.55-1.04)	0.084	0.76 (0.55-1.04)	0.084
Recessive	273 (92.86)	1415 (97.99)	<0.0001	**3.75 (2.11-6.68)**	**<0.0001**	**3.83 (2.15-6.82)**	**<0.0001**
Risk genotypes ^c^							
0	189 (64.29)	1123 (77.77)		1.00		1.00	
1-4	105 (35.71)	321 (22.23)	<0.0001	**1.94 (1.49-2.54)**	**<0.0001**	**1.94 (1.48-2.54)**	**<0.0001**

OR, odds ratio; CI, confidence interval; HWE, Hardy‒Weinberg equilibrium. Values were in bold if the 95% CIs excluding 1.00 or the *P* values less than 0.05. ^a^ χ^2^ test for genotype distributions between hepatoblastoma patients and cancer-free controls. ^b^ Adjusted for age and sex. ^c^ Risk genotypes were rs7641261 TT, rs2303476 CC, rs4257518 GG, and rs3762735 GG.

**Table 2:** Stratification analysis of the association between *TRMT10C* genotypes and hepatoblastoma susceptibility.

Variables	rs7641261 (cases/controls)		AOR (95% CI) ^a^	*P* ^a^	rs4257518 (cases/controls)		AOR (95% CI) ^a^	*P* ^a^	Risk genotypes (cases/controls)		AOR (95% CI) ^a^	*P* ^a^
	CC	CT/TT			AA	AG/GG			0	1-4		
Age, months												
<17	103/381	52/260	0.75 (0.52-1.08)	0.123	63/213	91/428	0.72 (0.50-1.04)	0.077	102/495	53/146	**1.78 (1.22-2.60)**	**0.003**
≥17	91/473	48/330	0.76 (0.52-1.10)	0.144	54/265	85/538	0.78 (0.54-1.13)	0.182	87/628	52/175	**2.13 (1.46-3.13)**	**0.0001**
Sex												
Females	77/344	43/251	0.77 (0.51-1.15)	0.198	44/200	75/395	0.86 (0.57-1.30)	0.482	76/450	44/145	**1.80 (1.19-2.73)**	**0.006**
Males	117/510	57/339	0.73 (0.52-1.04)	0.080	73/278	101/571	**0.67 (0.48-0.94)**	**0.021**	113/673	61/176	**2.05 (1.44-2.92)**	**<0.0001**
Clinical stages												
I+II	100/854	52/590	0.75 (0.53-1.07)	0.112	62/478	89/966	0.71 (0.51-1.00)	0.051	103/1123	49/321	**1.65 (1.15-2.38)**	**0.007**
III+IV	57/854	29/590	0.74 (0.47-1.17)	0.197	37/478	49/966	0.66 (0.42-1.02)	0.060	57/1123	29/321	**1.80 (1.13-2.87)**	**0.013**

AOR, adjusted odds ratio; CI, confidence interval. Values were in bold if the 95% CIs excluding 1.00 or the P values less than 0.05. ^a^ Adjusted for age and sex, omitting the corresponding stratification factor

## Abbreviations

m^1^A: N1-methyladenosine
TRMT10C: tRNA methyltransferase 10C
m^1^G: N^1^-methylguanine
BWS: Beckwith-Wiedemann syndrome
FAP: familial adenomatous polyposis
SNP: single-nucleotide polymorphism
m^6^A: N6-methyladenosine
m^7^G: N7-methylguanosine
MAF: minor allele frequency
LD: linkage disequilibrium
TFBS: transcription factor binding site
HWE: Hardy-Weinberg equilibrium
OR: odds ratio
CI: confidence interval
m^5^C: 5-methylcytidine
2′-O-Me: 2′-O-methylation
tDRs: tRNA-derived small RNAs
